# In Silico Binding Mode Analysis of *Blarina* Paralytic Peptides with the Human T-Type Ca Channel hCa_v_3.2

**DOI:** 10.3390/toxins17110549

**Published:** 2025-11-04

**Authors:** Nozomi Hara, Chihiro Sadamoto, Ryo Fukuoka, Yusuke Yano, Andres D. Maturana, Masaki Kita

**Affiliations:** 1 Department of Applied Biosciences, Graduate School of Bioagricultural Sciences, Nagoya University, Furo-cho, Chikusa, Nagoya 464-8601, Japan; 2 Promotion Office for Open Innovation, Institutes of Innovation for Future Society, Nagoya University, Furo-cho, Chikusa, Nagoya 464-8601, Japan

**Keywords:** *Blarina* paralytic peptides, synenkephalin, T-type Ca Channel, docking simulation

## Abstract

*Blarina* paralytic peptides (BPPs), neurotoxins from shrew saliva that paralyze mealworms, share high sequence similarity with human synenkephalin [1–53] (hSYN), a peptide released from proenkephalin together with opioid peptides that mediate analgesic and antidepressant effects in the brain. Both synthetic BPP2 and hSYN induce a hyperpolarizing shift in the human T-type voltage-gated calcium channel (hCa_v_3.2) at sub-micromolar concentrations, although only BPP2 causes paralysis in insects. To gain insight into the functions of these insectivorous animal-specific neurotoxins and the largely uncharacterized brain peptides, we investigated the structure prediction of BPPs and SYNs and their interactions with hCa_v_3.2. AlphaFold 3 modeling complemented available cryo-EM data and accurately reproduced the overall channel architecture; however, this inactivated-state model proved unsuitable for predicting agonistic binding of BPPs and SYNs. In contrast, docking simulations using an activated-state hCa_v_3.2 homology model revealed distinct ligand-dependent differences in binding energies, affinity, and conformational flexibility. Notably, the C-terminal tail of BPPs—particularly its variable length and flexibility—was identified as a key determinant for the interactions with the S4 voltage-sensing domain of the channel. These findings provide new insights into the evolutionary adaptation of venom peptides in mammals and into potential therapeutic strategies targeting neurological disorders.

## 1. Introduction

**Figure 1:**
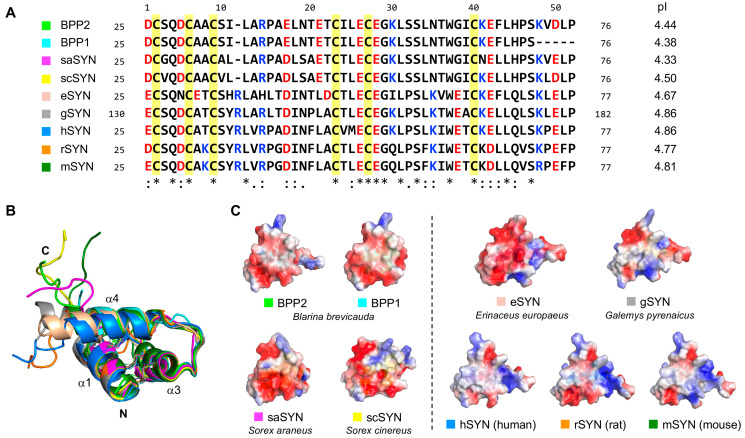
Structures of *Blarina* paralytic peptides (BPPs) and the mammalian synenkephalin N-terminal analogs (SYNs). (**A**) Sequence alignment of BPPs 2 and 1, saSYN (*Sorex araneus*), scSYN (*S. cinereus*), eSYN (*Erinaceus europaeus*), gSYN (*Galemys pyrenaicus*), hSYN (human), rSYN (rat), and mSYN (mouse). Asterisks indicate conserved residues among nine peptides, in which the six Cys residues are highlighted in yellow. Acidic and basic residues are shown in red and blue, respectively. The positions of BPPs (47 or 52 aa) and SYNs (52 or 53 aa) in proenkephalin (PENK) are indicated on either side of the sequence. Apparent pI (isoelectric point) values are shown to the right of the sequences. (**B**) Superposed predicted structures of BPPs and SYNs by ColabFold (BPP2 and hSYN) or AlphaFold 3 (seven other peptides) programs. Disulfide bonds are shown in stick models. (**C**) Surface charge models indicated by a red (negative)–blue (positive) heat map analysis.

The short-tailed shrew *Blarina brevicauda* is a rare venomous mammal, possessing potent neurotoxins in its saliva that allow to efficiently capture prey, such as earthworms and insects [1,2,3]. Since bites from *Blarina* shrews cause intense pain in humans [4], it has been suggested that their venom targets receptor channels involved in pain signaling and neurotransmission [5]. Early studies identified a mouse-lethal kallikrein-like protease, blarina toxin (BLTX) [6], along with its non-toxic homolog, blarinasin [7,8], from the salivary glands of *B. brevicauda*. Although these proteases showed little effect on invertebrates, we previously discovered *Blarina* paralytic peptides (BPPs) 1 and 2 as the principal paralytic components against mealworms (Figure 1A) [9]. BPP2 was synthesized by solid-phase peptide synthesis, native chemical ligation based on the precursor gene sequence of proenkephalin (PENK) [10]. We also established that BPP2 induces a hyperpolarizing shift (–11 mV) in the activation of the human T-type calcium channel (hCa_v_3.2) at 0.84 μM but does not have significant effect on N-type channel (hCa_v_2.2), an important calcium channel for neurotransmission and pain perception [11].

T-type calcium channels (Ca_v_3.1, 3.2, 3.3) are low-threshold voltage-gated channels characterized by rapid inactivation and slow deactivation [12]. These channels are widely distributed in the nervous, neuroendocrine, and cardiovascular systems, where they have important roles in sleep regulation, pain perception, and epileptogenesis [13,14,15]. Among them, Ca_v_3.2 is crucial for repetitive firing in both the brain and heart, and mutations in this channel are related to several syndromes, such as autism, epilepsy, Alzheimer’s disease, and amyotrophic lateral sclerosis [16,17,18]. Thus, identifying Ca_v_3.2-specific modulators and clarifying their gating and voltage regulation mechanisms may provide valuable opportunities for therapeutic development.

BPPs 1 and 2 have 47 and 52 amino acids, respectively, and contain six Cys residues to form three disulfide bonds. These sequences are highly similar to a segment of synenkephalin, a peptide expressed in the brain and other tissues, which correspond to the PENK 25–97 residues [19,20]. During PENK processing, synenkephalin is released together with Met-enkephalin and Leu-enkephalin [21]. The latter two act on opioid receptors in the brain and spinal cord, mediating analgesic and antidepressant effects [22]. Synenkephalin is abundant in the embryonic rat brain, where it may function in neuronal development [23], although its physiological role remains unresolved [24,25,26]. In addition, PENK processing and synenkephalin release have been observed in rat and human splenic mononuclear cells and bone marrow [27,28]. Meanwhile, synenkephalins have not been detected in the salivary glands of most mammals, except for certain shrews [10,29,30] and solenodons [31]. This result suggests that BPPs and SYNs are uniquely produced in saliva by insectivorous animals.

To enable direct comparison, we also synthesized the N-terminal fragment [1–53] of human synenkephalin (hSYN) as a human analog of BPPs, and investigated its structural stability, function, and biological relevance. hSYN displayed activation of the hCa_v_3.2 channel at 0.74 µM similar to BPP2, but it failed to cause paralysis in mealworms (*Zophobas atratus*) even at 10 times higher concentration than BPP2 (which is effective at 5.6 μg/g body weight). These results indicate that the paralytic effect of BPP2 in invertebrates is not directly attributable to hCa_v_3.2 channel activation.

The disulfide bond connectivity of BPP2 was determined to be Cys(I)–Cys(V), Cys(II)–Cys(IV), and Cys(III)–Cys(VI), by comparison of natural and synthetic peptides [9]. Synthetic hSYN displayed the same connectivity [32], whereas recombinant rat synenkephalin was reported to adopt an alternative pattern: Cys(I)–Cys(IV), Cys(II)–Cys(V), and Cys(III)–Cys(VI) [33]. To date, the disulfide bond pattern of native human brain synenkephalin (73 aa) remained unclear. However, we demonstrated that hSYN (53 aa), which shares the same C-terminal tail length as BPP2, preferentially adopts the thermodynamically stable disulfide bond pattern described above.

BPPs in *Blarina* shrew are thought to have evolved from non-toxic synenkephalins into neurotoxins capable of paralyzing invertebrate prey, with enhanced functionality emerging through adaptive modification. This evolutionary acceleration may account for the emergence of these peptides [34,35]. PENKs, which includes homologous sequences of BPPs, are expressed in the salivary glands of the common shrew (*Sorex araneus*) and the European water shrew (*Neomys fodiens*) [30]. Although these synenkephalins remain uncharacterized, they may serve as paralytic substances akin to BPPs, thereby conferring an ecological advantage in prey capture [36,37,38].

From this standpoint, structural comparison of mammalian SYNs and their binding modes with the hCa_v_3.2 channel provides insight not only into potential functions of poorly characterized brain peptides, but also into the chemical evolution of mammal-specific neurotoxins. In this study, we performed in silico binding mode analysis of BPPs and mammalian SYNs with hCa_v_3.2.

## 2. Results and Discussion

### 2.1. Sequence Alignment and Structure Comparison Among BPPs and SYNs

To compare the structures of BPPs and mammalian SYNs, we selected regions homologous to BPP2 from various mammalian PENKs. We aligned nine sequences, including BPP1, shrew-derived saSYN (*S. araneus*) and scSYN (*S. cinereus*), hedgehog-derived eSYN (*Erinaceus europaeus*), Pyrenean desman-derived gSYN (*Galemys pyrenaicus*), hSYN, rat-derived rSYN, and mouse-derived mSYN. The results showed that all six Cys residues (Cys2, Cys6, Cys9, Cys23, Cys27, and Cys40) as well as 10 residues (Gln4, Leu12, Glu26, Glu28, Gly29, Leu31, Ser33, Trp37, Leu44, and Ser47) are fully conserved (Figure 1A). Among SYNs, scSYN (81.1%) had the highest homology to BPP2, followed by saSYN (79.2%) and gSYN (60.4%), with overall homology ranging from 49% to 81%.

Next, to evaluate structural properties of BPPs and SYNs, these three-dimensional structures were constructed by AlphaFold 3 program [39]. This analysis suggested that BPP1 and SYNs all have helix-rich structures with the same disulfide bond connectivity as those in BPP2 and hSYN [9,32] previously prepared using a ColabFold program [40] (Figure 1B). The predicted N-terminal core structures of BPP1 and other SYNs including three major helices (α1, α3, α4) showed high similarity to BPP2 and hSYN, despite lacking the short α2 helix structure.

By substituting Ile11 on BPP2 with Tyr11-Arg12, hSYN has a polar interaction between Arg12 and Ser48 to stabilize the extended α4 helix at the C-terminus (Gln46–Ser48) [32]. Comparison with other species also revealed that shrew-derived BPP1, saSYN, and scSYN had a short α4 helix as with BPP2, and rSYN and mSYN had the similar middle-length structures with hSYN, while both eSYN and gSYN had a longer α4 helix extended to Glu51. Overall, the helices in the BPP and SYN models maintained nearly identical orientations despite slight differences in length, due to three conserved disulfide bonds, hydrophobic interactions between helices, and electrostatic interactions with polar residues, whereas the C-terminal random coil structure showed significant conformational diversity. The predicted template modeling (pTM) values of nine analogs were ranged in 0.57–0.72, with the helix structures relatively high confidence (Supplementary Figure S1A).

We further compared the surface charges of BPPs and SYNs, focusing on differences in acidic and basic residues (Figure 1C). The charged residues of BPP2, BPP1, saSYN, and scSYN were nearly identical, except for the Lys41Asn mutation in saSYN. The apparent pI values of these four shrew-derived peptides ranged from 4.33 to 4.50, whereas those of other SYNs were relatively high (4.67–4.86) due to the insertion of Arg12 and the mutation of Asn35 to Lys36.

The C-termini of the above-mentioned SYNs are shorter than those of synenkephalins that exist in the brain and other organs of humans, rats, and mice, and their mature structures remain unclear. Still, our results suggest that variations in the length of the α4 helix and the conformation of the random coil structure in the C-terminal tail have arisen as the result of SYN sequence mutation during mammalian evolution. These differences have important implications for the generality and specificity of the biological activities of BPPs, such as ion channel activation and paralysis in mealworms.

### 2.2. Construction of the Human T-Type Calcium Channel (hCa_v_3.2) Models

Voltage-gated ion channels undergo large structural changes between the inactivated (close), activated (open), and resting states. Recently, cryo-electron microscopy (cryo-EM) structures of human T-type calcium channels (hCa_v_3.1 [41], hCa_v_3.2 [16], and hCa_v_3.3 [42]) have been shown in antagonist-bound or apo forms, but none as the activated state. The core structures of Ca_v_ channels that include pore domains I–IV (PD_I–IV_, with S5 and S6 helices) and voltage-sensing domains I–IV [VSD_I–IV_, with S1–S4 transmembrane (TM) helices]—closely resemble that of Na_v_ channels [43]. The clockwise arrangement of the four homologous repeats when viewed from the extracellular side is common to all human Ca_v_ and Na_v_ channels.

To investigate the effects of BPP2 and hSYN, we previously constructed an activated hCa_v_3.2 channel model by homology modeling, using the activated human Na_v_1.4 channel (PDB: 6AGF) [44] as a template, and conducted PPI-docking studies [32]. However, this model lacked portions of the extracellular loop (ECL) and cytoplasmic helix (CH) domains, due to their low sequence similarity with the template and absence in available cryo-EM structures.

**Figure 2:**
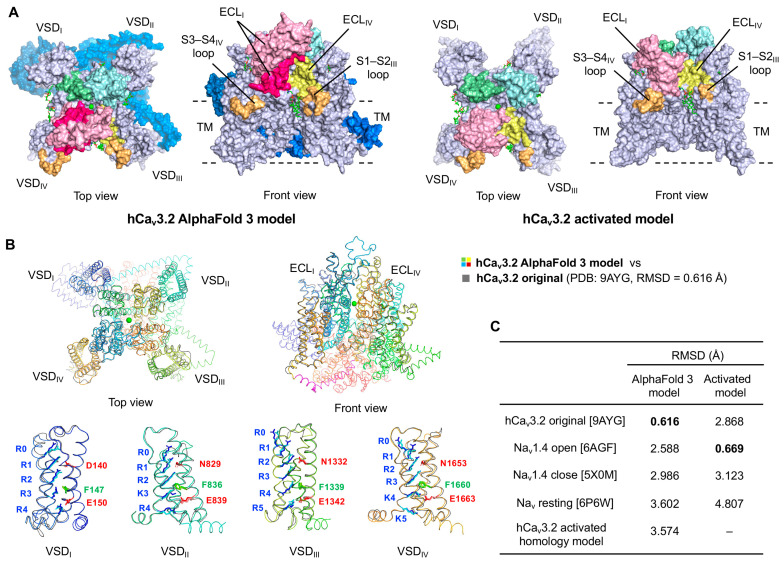
Proposed structures of hCa_v_3.2 channels. (**A**) Structures of AlphaFold 3-predicted and the activated channel homology models. The S1–S2/S3–S4 loops and extracellular loop (ECL) domains I–IV are highlighted in multi-pastel colors. The ECL_I_ and cytosolic domains complemented in the AlphaFold 3 model are highlighted in hot pink and marine, respectively. (**B**) Superposed structure of AlphaFold 3 model (multicolor) with the original cryo-EM hCa_v_3.2 channel (PDB: 9AYG, gray). The S3–S4 linker is shown in magenta. Characteristic gating positively charged residues of the S4 helix and their opposing residues in the adjacent S2 and S3 helices are highlighted in the stick models. (**C**) RMSD values of the hCa_v_3.2 AlphaFold3 and activated [32] models compared with the original hCa_v_3.2 and template Na_v_ channels. Each PDB ID is shown in square brackets.

In the present study, to enable more accurate PPI-docking analyses of BPPs and SYNs, we employed the full-length sequence of hCa_v_3.2 for structure prediction using AlphaFold 3. This yielded a properly folded, full-length hCa_v_3.2 channel model, as the rank 2 model (Figure 2A, left). The interface predicted template modeling (ipTM) and pTM scores of the AlphaFold 3 model were 0.98 and 0.62, respectively, with high confidence in TM and the ECLs of VSD (Supplementary Figure S1B). When superimposed on the cryo-EM original structure of hCa_v_3.2, the AlphaFold 3 model displayed a high degree of similarity (RMSD 0.616 Å) (Figure 2B). The positions of the positively charged gating residues R0–R5 (or K3–K5) in the S4 helix, and the counter-residue in the near S2 and S3 helices forming the charge transfer center, are crucial for the channel activation in response to membrane depolarization. In the VSD_I_ of the AlphaFold 3 model, Phe147 was located near R3, and Asp140 and Glu150 formed electrostatic interactions with R1 and R4, respectively, consistent with the original hCa_v_3.2 structure. Similarly, in the VSD_II~IV_, the corresponding positively charged residues were positioned almost identically to those in the cryo-EM hCa_v_3.2 structure.

By contrast, structural identity between the AlphaFold 3 model and the activated hCa_v_3.2 channel homology model [32] (Figure 2A, right), as well as the three Na_v_ channels, was relatively low (RMSD 2.588–3.602 Å) (Figure 2C). Therefore, we performed PPI-dock of BPPs and SYNs using the AlphaFold 3 model, which includes the previously missing ECL and CH domains, and compared these results with those obtained from the activated-state model.

### 2.3. PPI-Dock Models of the hCa_v_3.2–BPP and SYN Complexes

We previously carried out protein–protein interaction docking (PPI-Dock) simulations using the S1–S2 and S3–S4 ECLs of VSD_I–IV_ in the activated hCa_v_3.2 model as ligand-binding sites [32], according to the related gating-modifier toxins. In fact, both BPP2 and hSYN were localized between ECL_I_ and ECL_IV_ with the best S scores (lowest in energy). In this simulation, we designated only the two VSD loops (S1–S2_III_ and S3–S4_IV_) together with the adjacent ECL_I_ and ECL_IV_ as binding sites and performed PPI-Dock analyses of BPP and SYN against two hCa_v_3.2 channel models.

**Figure 3:**
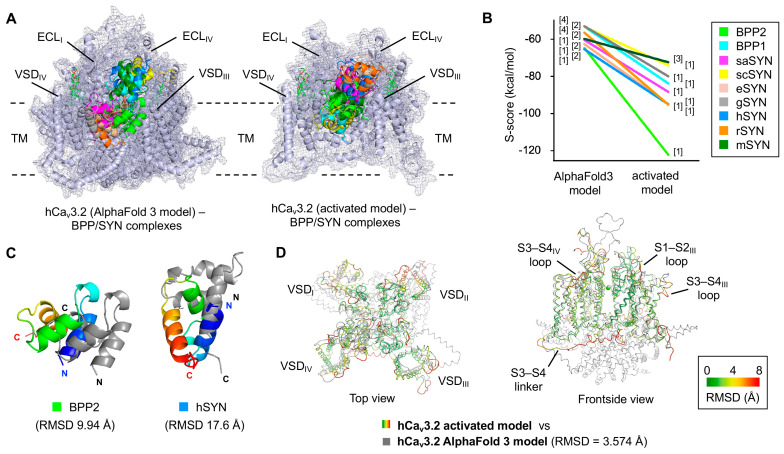
Posing and scoring of the docking models of hCa_v_3.2–BPP/SYN complex. (**A**) Superimposed structures of the hCa_v_3.2 AlphaFold 3 or activated models with BPPs and SYNs, selected from those with the lowest docking scores bound to the ECL_I_–ECL_IV_ sites. (**B**) Comparison of the S-scores for the two hCa_v_3.2 models shown in (**A**). Each pose ranking is shown in square brackets. (**C**) Superimposed structures of BPP2 and hSYN on the two hCa_v_3.2 models in (**A**) (from the front views). (**D**) Superimposed structures of the activated hCa_v_3.2 model (multicolor based on RMSD values) with the AlphaFold 3 model (gray).

In the activated hCa_v_3.2 model, all ligands except mSYN were located to the ECL_I-IV_ domain in the top-ranked pose (Figure 3A, right). The complexes with BPP2 and hSYN reproduced our previous docking results [32] with high consistency (S scores –122.07 and –94.90 kcal/mol, respectively) and were more stable than those with almost all other ligands (S scores –95.04 to –72.28 kcal/mol) (Figure 3B). In contrast, the AlphaFold 3 model favored binding sites distal to the ECLs, such as within TM site or outside the VSD, to select the ranked 2 and 4 poses (Figure 3A, left). This model also preferred BPP2 and hSYN (S scores –64.61 and –65.28 kcal/mol, respectively), producing ranking similar to the activated model (S scores –62.49 to –52.84 kcal/mol for others). However, all AlphaFold 3 complexes were less stable overall, and the binding energy differences among ligands were narrower compared to the activated model.

Regarding specific binding modes, BPP2 bound to hCa_v_3.2 channel in nearly the same orientation in both the activated and AlphaFold 3 models but exhibited substantial differences in distance (RMSD ~10 Å) (Figure 3C). The binding modes of hSYN and other ligands varied even more markedly. Thus, we concluded that the PPI-Dock using the AlphaFold 3 model yielded low accuracy, similar to results with the hCa_v_3.2 cryo-EM structure.

When the two hCa_v_3.2 models were superimposed to examine local structural variation, the VSD TM regions overlapped well, but significant differences were observed in the S1–S2 and S3–S4 loops of VSD_I–IV_, which likely underlie the differences in ligand interactions and binding affinities between the AlphaFold 3 and activated models (Figure 3D). The S3–S4 linker, which undergoes large conformational shifts during activation, inactivation, and resting, also showed markedly different orientations between the two models. Therefore, the apo-AlphaFold 3 model, which closely resembled the hCa_v_3.2 cryo-EM structure, proved unsuitable for analyzing agonistic binding modes of BPP2 and hSYN. While it is reasonable that hSYN binds more strongly to the activated hCa_v_3.2 channel than other mammalian SYNs, it is noteworthy that BPP2, derived from the venom of the *Blarina* shrew, a species with a particularly potent venom, bound even more strongly.

**Figure 4:**
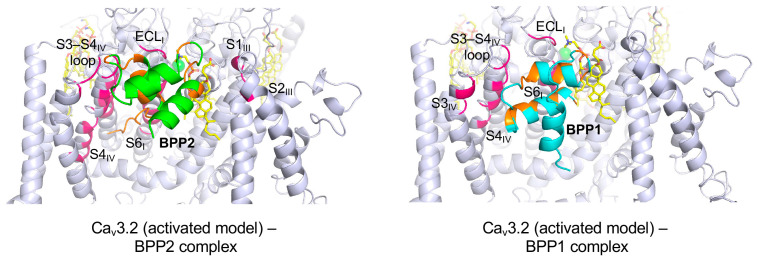
Detailed views of the interaction between the activated hCa_v_3.2 channel and BPP2/BPP1 obtained by the PPI-Dock simulations. The interacting residues between channel and peptide ligands are highlighted in hot pink and orange, respectively.

As shown in Figure 3A,B, BPP2 and BPP1 exhibited significant differences in both binding energy and binding site within the activated hCa_v_3.2 model. To clarify why their difference of just five C-terminal residues producing such striking functional divergence, we analyzed their specific PPIs (Figure 4). The C-terminal tail of BPP2 interacted with multiple hydrophobic residues within the S4_IV_ domain, whereas BPP1 bound to the same region through its α1- and α2-helices (Supplementary Figure S2). In the activated hCa_v_3.2 model, BPP2 interacted with 32 residues, compared to only 17 residues for BPP1, a difference likely to have major impact on binding stability. These results proposed that the variable length and flexibility of the C-terminal tail in BPP2 may affect their unique and effective interaction with hCa_v_3.2.

## 3. Conclusions

Focusing on the mechanism by which the shrew-derived paralytic peptide BPP2 activates the human T-type calcium channel hCa_v_3.2, we predicted the three-dimensional structures of BPPs and SYNs, N-terminal homologs of mammalian synenkephalins. Peptide modeling revealed differences in the α4 helix length and the conformational flexibility of the C-terminal tail. The AlphaFold 3 model examined here complemented the hCa_v_3.2 cryo-EM structure and provided accurate predictions of the overall channel architecture. However, because this model represents the apo and inactive state, it was unsuitable for accurately predicting binding to BPPs and SYNs that act as channel activators. In contrast, PPI-Dock simulations using the activated hCa_v_3.2 model revealed marked differences in binding energies, reflecting ligand-dependent variations in affinity and conformation that could not be explained by sequence similarity alone.

Our results provide an important basis for the unique biological activity of BPP2, which has independently evolved as a mammalian venom peptide and whose fragment peptides contribute to paralysis, unlike other mammalian peptides. Although further validation of docking models by molecular dynamics simulations and detailed structure–activity studies of BPP1 and SYNs are required, our findings highlight the importance of the C-terminal tail—particularly its variable length and flexibility—in mediating PPIs with the VSD and modulating the S4 voltage-sensing domain. Looking ahead, we plan to employ photoaffinity and ligand-dissociation probes [45,46] of BPPs to clarify the activation mechanism of hCa_v_3.2 in more detail, and to assess their interactions with other transmembrane receptors, such as transient receptor potential (TRP) channels, as well as the resulting physiological responses. These efforts aim to elucidate the distinctive neurotoxic functions of BPPs and analyze their binding sites with unknown molecular targets in the nervous system.

## 4. Materials and Methods

### 4.1. Data Source

The amino acid sequences and disulfide bond pattern of BPPs 1 and 2 were determined by the spectroscopic analysis and degradation of natural and synthetic peptide toxins [9]. The mammalian SYN sequences used were the precursor PENK sequence registered in the gene database (Table 1).

### 4.2. Molecular Modeling Studies

Three-dimension structures of BPPs and SYNs were constructed by using ColabFold [40] (a Google Colab version of AlphaFold2 [53] using MMSeq2 [54]) (for BPP2 [9] and hSYN [32]) or AlphaFold 3 [39] (for BPP1 and other SYNs), stereostructure prediction programs based on the amino acid sequences. The most confident model (rank 1, unless otherwise noted) for each calculation was used for structural analyses and figure representation.

Three-dimensional structures of the human T-type calcium channel (hCa_v_3.2) were predicted using AlphaFold 3, and the rank 2 model with a properly constructed secondary structure was used for further analysis (The rank 1 model was not selected due to the lack of appropriate secondary structure throughout the sequence). The activated hCa_v_3.2 channel model was obtained by homology modeling using human Na_v_1.4 (PDB: 6AGF) as a template, as described previously [32]. Docking simulation was manually performed in protein–protein interaction docking (PPI-Dock) mode using the Molecular Operating Environment (MOE) 2022.02 program package (Chemical Computing Group, Inc., Montreal, Canada) [55,56]. For docking model studies, phospholipids and Ca^2+^ ions present in the original cryo-EM structure of Ca_v_3.2 channel (PDB: 9AYG) were added to the two models obtained.

Conformational searches were performed using the Amber14:EHT force-field with GB/VI Generalized Born implicit solvent electrostatics (*D*_in_ = 1, *D*_out_ = 80) and with LowModeMD [9,32]. Protonation states of ligands and receptors were assigned at pH 7.0 using default parameters of QuickPrep module in MOE. The S1–S2_III_ and S3–S4_IV_ loops on the Ca_v_3.2 channel, and the adjacent ECL_I_ and ECL_IV_ domains, were designated as the ligand-binding sites. The docking boxes used corresponded to a 54 × 52 × 32 Å bounding box for the AlphaFold 3 model and a 56 × 46 × 19 Å bounding box for the activated hCa_v_3.2 homology model (missing part of the adjacent ECL_I_ domain), respectively. From the 10,000 generated poses, refinement was performed using rigid-body models (induced-fit model or side-chain flexibility analysis were unavailable due to the large molecular weights of the ligands), and the top 300 minimized binding poses were prioritized based on the S values, which indicate the binding stability between channels and peptide ligands. The lowest-energy (rank 1) (or 2nd–4th lowest, ranks 2–4) conformations among the models bound between the ECL_I_ and ECL_IV_ sites of BPPs and SYNs were compared for binding energy and modes. PyMol was used for the PPI analysis and final image processing suitable for scientific representation. MOE was used to color and visualize the RMSD values for each residue.

## Figures and Tables

**Table 1:** Data source of BPPs and SYNs from proenkephalin (PENK) precursors.

Name	Organism	Locus (Accession No.)	Reference
BPP1/2	*Blarina brevicauda*	MT559766	[10]
saSYN	*Sorex araneus*	NC_073303	– ^1^
scSYN	*Sorex cinereus*	NW_026606059	– ^1^
eSYN	*Erinaceus europaeus*	NW_006804460	– ^1^
gSYN	*Galemys pyrenaicus*	JAGFMF010011524	[47]
hSYN	*Homo sapiens*	PENK_HUMAN (P01210)	[38,48]
rSYN	*Rattus norvegicus*	AH002996	[49,50,51]
mSYN	*Mus musculus*	NP_001335138	[52]

^1^ Not available.

## Data Availability

The data presented in this study are available in [NAGOYA Repository] at [https://nagoya.repo.nii.ac.jp/records/2013372, (accessed on 31 October 2025)], reference number [10.18999/2013372]. These data were derived from the following resources available in the public domain: [https://doi.org/10.18999/2013372].

## Supplementary Materials

The following supporting information can be downloaded at: https://www.mdpi.com/article/10.3390/toxins17110549/s1, Figure S1: Calculated pLDDT and PAE (predicted aligned errors) confidence plots of generated by Colabfold (BPP2 and hSYN) or AlphaFold 3 (other seven peptides and Ca channel) programs. (A) BPPs and SYNs. (B) hCa_v_3.2.; Figure S2: Interacted residues on the activated hCa_v_3.2 channel and the ligands (BPP2 and BPP1) on the complexes are shown in *. The residues of S1–S2 and S3–S4 loops and the VSD residues in hCa_v_3.2 channel are shown in magenta and cyan, respectively.
