# Supplementary material for: In Silico Binding Mode Analysis of Blarina Paralytic Peptides with the Human T-Type Ca Channel hCav3.2
Source: Toxins (Basel). 2025 Nov 4;17(11):549. doi: 10.3390/toxins17110549 (PMC12656432; doi:10.3390/toxins17110549)
Supplement: Supplementary file 1 [file toxins-17-00549-s001.zip › toxins-3929553-supplementary.pdf]

Supplementary Materials

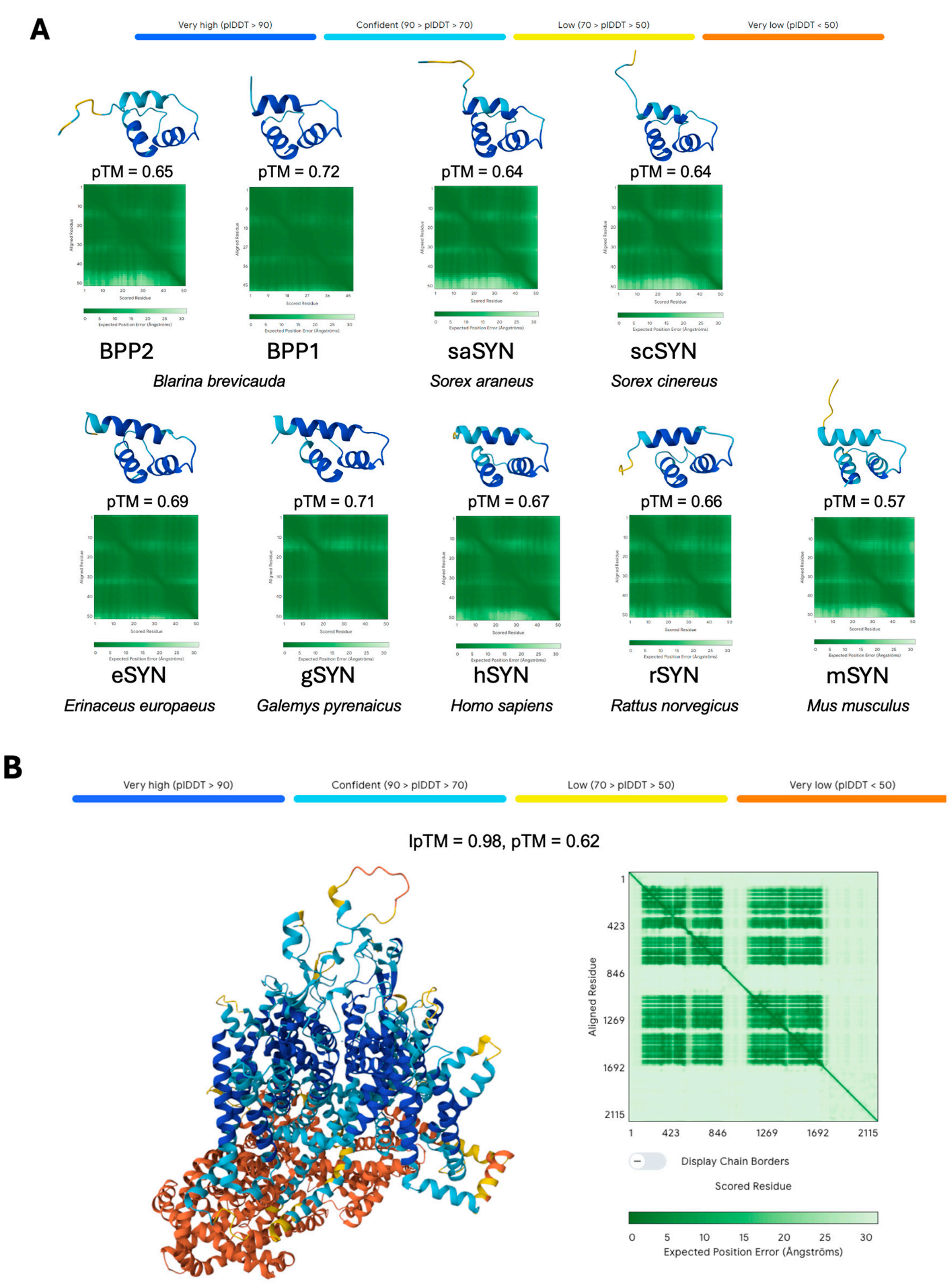

Figure S1. Calculated pLDDT and PAE (predicted aligned errors) confidence plots of generated by Colabfold (BPP2 and hSYN) or AlphaFold 3 (other seven peptides and Ca channel) programs. (**A**) BPPs and SYNs. (**B**) hCa$_v$3.2.

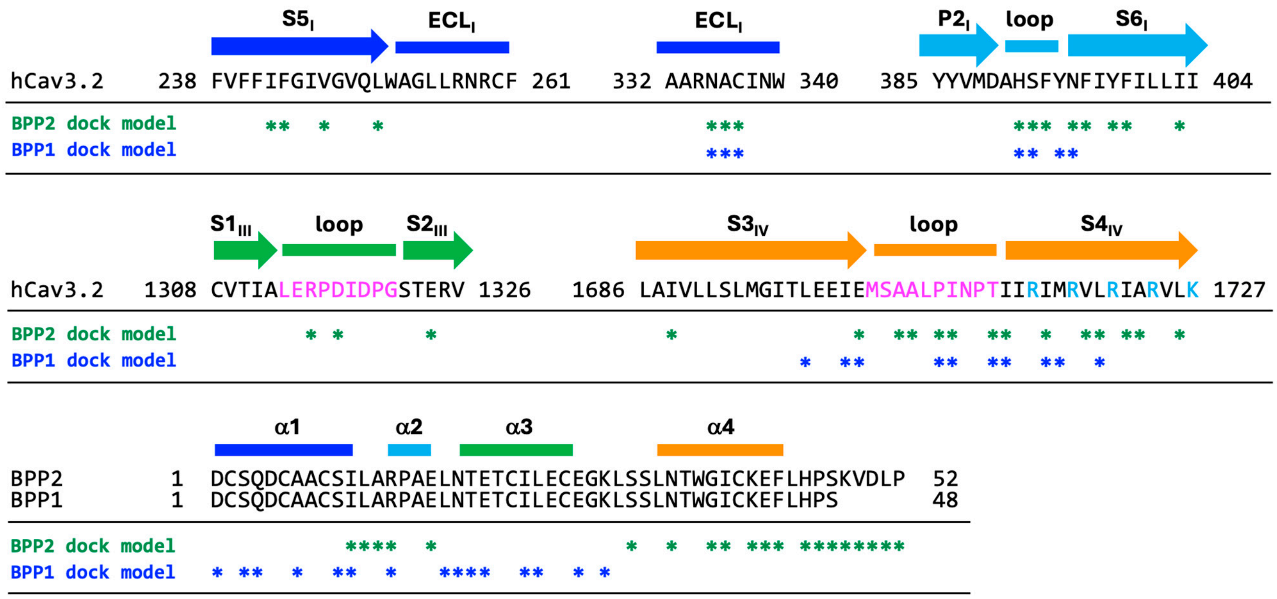

Figure S2. Interacted residues on the activated hCa$_v$3.2 channel and the ligands (BPP2 and BPP1) on the complexes are shown in *. The residues of S1–S2 and S3–S4 loops and the VSD residues in hCa$_v$3.2 channel are shown in magenta and cyan, respectively.
